# Partial Methylation at Am100 in 18S rRNA of Baker's Yeast Reveals Ribosome Heterogeneity on the Level of Eukaryotic rRNA Modification

**DOI:** 10.1371/journal.pone.0089640

**Published:** 2014-02-28

**Authors:** Markus Buchhaupt, Sunny Sharma, Stefanie Kellner, Stefanie Oswald, Melanie Paetzold, Christian Peifer, Peter Watzinger, Jens Schrader, Mark Helm, Karl-Dieter Entian

**Affiliations:** 1 DECHEMA Research Institute, Biochemical Engineering, Frankfurt am Main, Germany; 2 Institute for Molecular Biosciences, Johann-Wolfgang Goethe University, Frankfurt am Main, Germany; 3 Institute of Pharmacy and Biochemistry, Johannes Gutenberg University, Mainz, Germany; The John Curtin School of Medical Research, Australia

## Abstract

Ribosome heterogeneity is of increasing biological significance and several examples have been described for multicellular and single cells organisms. In here we show for the first time a variation in ribose methylation within the 18S rRNA of *Saccharomyces cerevisiae*. Using RNA-cleaving DNAzymes, we could specifically demonstrate that a significant amount of *S. cerevisiae* ribosomes are not methylated at 2′-*O*-ribose of A100 residue in the 18S rRNA. Furthermore, using LC-UV-MS/MS of a respective 18S rRNA fragment, we could not only corroborate the partial methylation at A100, but could also quantify the methylated versus non-methylated A100 residue. Here, we exhibit that only 68% of A100 in the 18S rRNA of *S.cerevisiae* are methylated at 2′-*O* ribose sugar. Polysomes also contain a similar heterogeneity for methylated Am100, which shows that 40S ribosome subunits with and without Am100 participate in translation. Introduction of a multicopy plasmid containing the corresponding methylation guide snoRNA gene SNR51 led to an increased A100 methylation, suggesting the cellular snR51 level to limit the extent of this modification. Partial rRNA modification demonstrates a new level of ribosome heterogeneity in eukaryotic cells that might have substantial impact on regulation and fine-tuning of the translation process.

## Introduction

Ribosomes are cellular organelles essential to all known forms of life on earth. In all organisms these macromolecular machines achieve the translation of the genetic code into proteins. All ribosomes are composed of two subunits, each of them consisting of at least one rRNA molecule and several ribosomal proteins. Although function and overall structure of these complexes are highly conserved during evolution, their composition varies from species to species. Whereas the small subunit of *Escherichia coli* contains 21 ribosomal proteins, the human counterpart consists of 33 proteins [1]. And although most cell biology textbooks still give the impression that an organism contains one type of ribosome that is composed of clearly defined parts, the concept of ribosome heterogeneity has been discussed for quite a long time. The first review on this topic, prepared by S. Ramagopal [2], summarized the first reports describing non-uniformity of ribosomes in one species. The earliest hints for the occurrence of slightly different ribosomal proteins in different organs of rabbit were published by Delaunay et al. [3]. Also using two-dimensional gel electrophoresis Lambertsson [4] could detect developmental stage-specific ribosomal proteins in *Drosophila melanogaster* cells.

Following reports, also discussed by Ramagopal [2], showed other examples of ribosomal protein heterogeneity but also ribosome heterogeneity at the level of rRNA sequence. The most exciting finding was the rather specific synthesis of a distinct type of 18S rRNA in different life cycle stages of the protozoan parasite *Plasmodium berghei* [5]. Two recently published reviews summarize many more examples for ribosome heterogeneity on the level of ribosomal proteins and rRNA sequence and also discuss physiological functions of changes in ribosome composition [6], [7]. Both reviews also speculate about post-transcriptional modifications as a source of ribosomal diversity for more specialized functions in eukaryotic cells. *S. cerevisiae* rRNAs contain 44 pseudouridines (Ψ), 54 2′-*O*-ribose methyl groups and nine base modifications [8], [9]. Whereas base modifications are introduced by specific enzymes, the pseudouridinylations and 2′-*O*-ribose methylations are carried out by small nucleolar ribonucleoprotein particles (snoRNPs), each containing a modification site-specifying snoRNA. Interestingly, Gilbert [6] as well as Xue and Barna [7] reviewed data which might contradict the prevailing idea of snoRNAs playing constitutive roles in ribosome biogenesis, and that all rRNA target sites are fully modified under all conditions. Heterogeneous snoRNA expression levels in different tissues [10] and oscillation of the expression of some snoRNAs in a circadian manner [11] further supported their notion. Additionally, knockdown studies using morpholino oligomers showed that depletion of different snoRNAs in zebra fish causes specific developmental phenotypes [12], accentuating highly specific and significant effects of particular rRNA modifications. For *S. cerevisiae*, Esguerra uncovered an unexpected rich phenotypic diversity of different snoRNA gene deletion mutants and also proposed the existence of rRNA modification heterogeneity [13], [14]. He went even further and suggested the direct participation of snoRNAs in cellular stress responses with compositionally distinct ribosomes that are customized according to environmental cues. Partial rRNA modifications were found in the prokaryotes *Escherichia coli* [15], *Bacillus subtilis* and *Sulfolobus acidocaldarius* [16]. For two adenocarcinoma cell lines significant increases in the 2′-*O*-ribose methylation level at some sites could be shown in the more aggressive cancerous cells, which already supports the existence of partial rRNA modification at specific positions under nonpathological situations [17].

Although their existence has been assumed many times, it has to our knowledge not been demonstrated that certain heterogeneities with respect to the snoRNA-mediated rRNA modifications exist in eukaryotic cells under physiological conditions, not to mention develop as response to environmental perturbations. Here we show for the first time that a substantial amount of ribosomes in actively growing *S. cerevisiae* cell culture lacks a specific snoRNA-mediated modification, namely the 2′-*O*-ribose methyl group at nucleotide A100 in the 18S rRNA.

## Results and Discussion

### 18S rRNA of *S. cerevisiae* contains partial 2′-*O*-ribose methylation at A100

**Figure 1:**
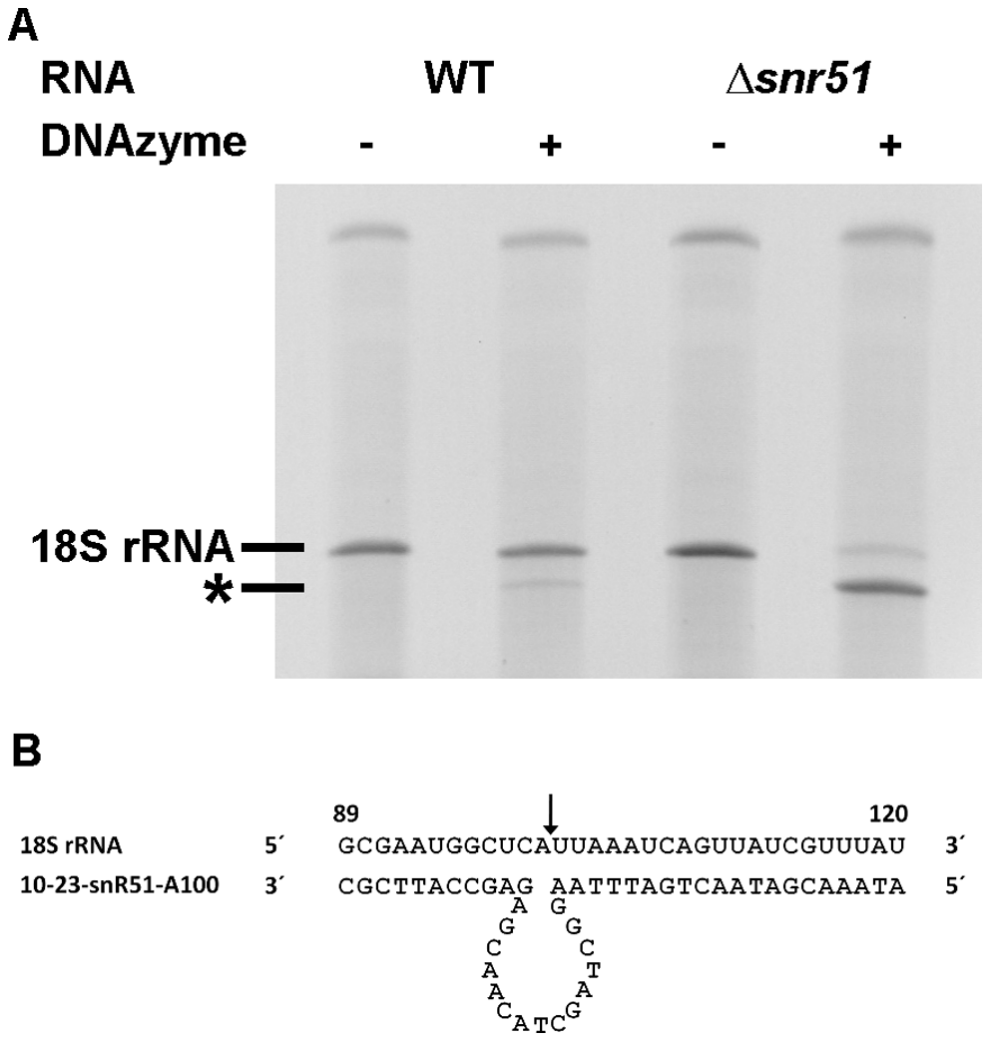
Identification of 18S rRNA molecules in wild type (WT) and Δ*snr51* that lack the 2′-*O*-ribose methyl group at nucleotide A100. A) Detection of 18S rRNA molecules lacking the Am100 modification by DNAzyme cleavage. Total RNA from wild type and Δ*snr51* cells grown in YEPD medium to exponential phase was incubated with or without the DNAzyme 10-23-snR51-A100 and afterwards analyzed by gel analysis. The RNA band marked with an asterisk represents the large 18S rRNA fragment after cleavage at nucleotide A100. The 100 nucleotide fragment ran out of the gel. B) Schematic illustration of DNAzyme 10–23-snR51-A100 binding to its target site in 18S rRNA. The cleavage site 3′ to A100 is marked with an arrow. The resulting fragments have lengths of 100 nt and 1700 nt.

We previously demonstrated that RNA-cleaving DNAzymes can be used to detect 2′-*O*-ribose methylated nucleotides in RNA molecules in a very simple and straightforward way [18]. DNAzyme-mediated site-specific scission of an RNA molecule at a given position is impeded by a 2′-*O*-ribose methyl group at the nucleotide 5′ to the cleavage site. During our analysis of different DNAzymes for the investigation of several modified nucleotides in *S. cerevisiae* rRNAs, we observed an unexpected result for one ribose methylation, catalysed by snR51 snoRNP. snR51 catalyzes the 2′-*O* ribose methylation of adenosine (Am) at position 100 in the 18S rRNA of *S.cerevisiae*. Intriguingly, when the DNAzyme 10–23-snR51-Am100, specific for the Am100 was used, we could not only detect the cleavage product with an RNA sample from the Δ*snr51* strain, but also with RNA from the respective wild type strain CEN.PK2-1C (figure 1). To rule out the possibility that this phenomenon is unique to the CEN.PK strain we also tested the lab strain BY4741, a baker's yeast strain isolated from cider (SC-F3-1) and a *Candida glabrata* strain. All these microorganisms contained a comparable amount of 18S rRNA devoid of 2′-O ribose methylation at residue A100 (data not shown). This unambiguously demonstrates the existence of a significant amount of ribosomes in yeast cells that do not contain this snoRNA-mediated modification of the 18S rRNA. To our knowledge this is the first report of ribosome heterogeneity regarding snoRNA-mediated rRNA modifications.

### Quantification of the amount of Am100 methylation in wild type 18S rRNA

**Figure 2:**
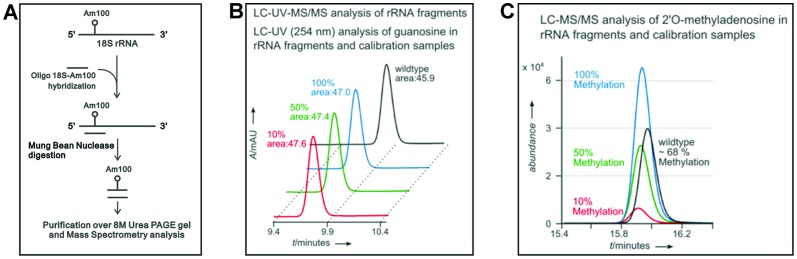
LC-UV-MS/MS analysis of the isolated rRNA fragment and calibration samples. (A) Schematic diagram for the mung bean nuclease method, used here to isolate the 18S rRNA fragment containing Am100 residue for subsequent mass spectrometry analysis. (B) UV chromatograms of all 4 samples and peak areas. In red, the sample containing 100% guanosine and 10% 2′-*O*-methyladenosine is shown. In green and blue the respective 50% and 100% Am turnover samples are shown. The black chromatogram shows the guanosine peak of the rRNA fragment. (C) Overlay of MS/MS chromatograms for 2′-*O*-methyladenosine. The methylation extent of the rRNA fragment was found to be 68% as could be seen in comparison to the calibration samples.

To further augment the partial ribose methylation at A100 and quantify the exact amount of Am100 ribose methylation in 18S rRNA of the wild type, we isolated the fragment containing the Am100 residue from wild type 18S rRNA by Mung bean nuclease protection method [9] (figure 2A).

The isolated rRNA fragment was then subjected to LC-UV-MS/MS analysis. For quantification of the 2′-*O*-methyladenosine level in the sample, two approaches were used as described in Materials and Methods. Figure 2 shows the results of the sample measurement in comparison to three calibration measurements. The calibration samples contained the same amount of guanosine as the analyzed RNA fragment, observed as similar peak areas (figure 2B and C). However, the amount of 2′-*O*-methyladenosine varies in the calibration samples from 10% to 100% compared with the guanosine level. As evident in figure 2C, the peak area of the RNA fragment was in between the 50% and 100% calibration sample. With the help of an internal standard, the amount of 2′-*O*-methyladenosine was found to be 68% (raw data and applied calculations can be found in the supplementary information).

### Ribosomes lacking the Am100 modification in the 18S rRNA participate in translation

**Figure 3:**
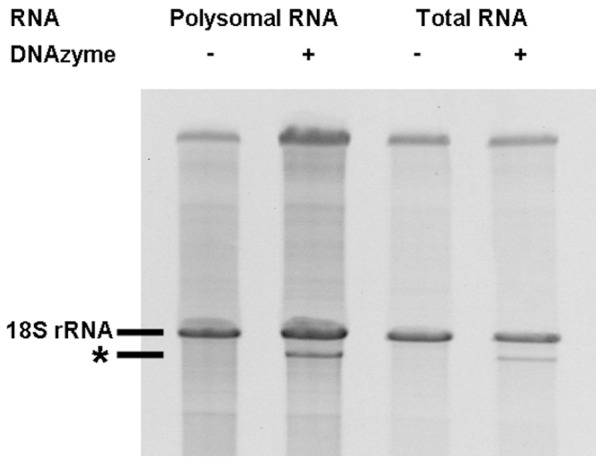
Comparison of 18S rRNA molecules lacking the 2′-*O*-ribose methyl group at nucleotide A100 in polysomal RNA and total RNA. Polysomal RNA and total RNA isolated from wild type cells that were grown in YEPD medium to exponential phase was incubated with or without the DNAzyme 10–23-snR51-A100 and afterwards analyzed by gel analysis. The RNA band marked with an asterisk represents the large 18S rRNA fragment after cleavage at nucleotide A100.

Although it can be assumed that in cells from the logarithmic growth phase used here, most 18S rRNA molecules are part of actively translating ribosomes, the possibility exists that a certain population are a constituent of immature or inactive ribosome forms. In order to find out if ribosomes without the 2′-*O*-ribose methyl group at A100 in the 18S rRNA are such nonfunctional specimens, we analyzed RNA from purified polysomes. As evident from figure 3, our results clearly show that the ratio of 18S rRNA containing Am100 *versus* unmethylated A100 18S rRNA was the same as found in total RNA. This experiment proved that ribosomes without the methylation at A100 are actively engaged in protein synthesis.

### Extent of A100 modification is probably limited by the amount of snR51

**Figure 4:**
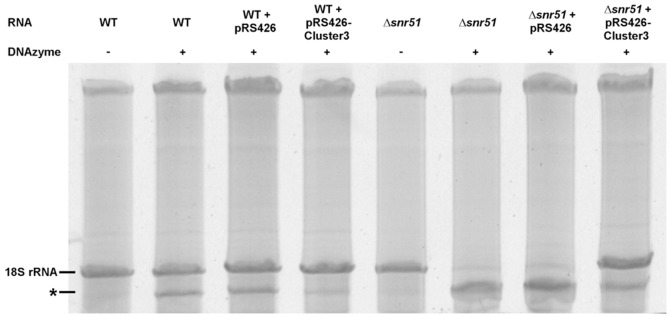
Investigation of changes in Am100 modification extent in 18S rRNA after introduction of a multicopy plasmid containing *SNR51*. Total RNA isolated from wild type and Δ*snr51* cells grown in synthetic medium to exponential phase containing no plasmid, the plasmid pRS426 or the plasmid pRS426-Cluster3 was incubated with the DNAzyme 10–23-snR51-A100 and afterwards analyzed by gel analysis. The RNA band marked with an asterisk represents the large 18S rRNA fragment after cleavage at nucleotide A100.

After demonstrating ribosome heterogeneity in *S. cerevisiae* on the level of rRNA modification, we addressed possible reasons for the phenomenon. First we hypothesized that two snoRNA-mediated modifications (Ψ106 and Ψ120) in close proximity to A100 might lead to spatial hindrance and impair the efficiency of 2′-*O*-ribosome methylation at A100 during the maturation process. A very similar situation regarding the spatial clustering of rRNA modifications exists in case of the snR40-mediated 2′-*O*-ribose methylation of G1271. However, despite the adjacent modifications at U1269 and U1290, no molecules lacking the methylation at position 1271 could be detected with a highly efficient DNAzyme using our method (data not shown). This finding also excludes that snoRNAs responsible for modifications at several target sites always lead to only partial modification of rRNAs in the cell, as both snR51 and snR40 target one nucleotide in the 18S rRNA and one nucleotide in the 25S rRNA. This observation supported our view that the lack of A100 methylation in some ribosomes is no inevitable consequence of the target nucleotide position but a fine-tuned state evolved during evolution. The intention of our next experiment was therefore to test the possibility of achieving a more complete modification at this position. For that purpose a multicopy plasmid containing the snoRNA gene cluster 3 encoding snR51, snR70 and snR41 (pRS426-Cluster3) was constructed and transformed into wild type and Δ*snr51* strain. The results obtained after DNAzyme analysis of RNA samples from the resulting strains are shown in figure 4. Introduction of the vector into the Δ*snr51* strain led to a strong recurrence of modified and hence non-cleavable 18S RNA, demonstrating the functionality of the construct. The presence of pRS426-Cluster3 in wild type indeed resulted in a clear increase of 2′-*O*-ribose methylation at A100. This finding suggests that it is apparently the amount of snR51 that limits the extent of A100 modification in the 18S rRNA of bakers yeast.

Given the heterogeneity of cellular yeast ribosomes with regard to this specific rRNA modification to be a precisely adjusted state, the question arises what special function do the ribosomes lacking this modification might have. Up to now no significant growth disadvantage of the Δ*snr51* strain or the *SNR51*-overexpression strain compared to the wild type could be detected. This is however not surprising as single modification guide snoRNA gene deletion mutants exhibit in most cases only marginal growth phenotypes under standard growth conditions but show altered growth dynamics under a wide array of environmental perturbations, including ribosomal antibiotics [14]. Besides transcriptome, proteome or metabolome analysis other promising approaches to uncover functional necessities of this type of ribosome heterogeneity are the use of high-resolution phenotyping [14] as well as genetic arrays [19]. For all approaches in this direction it is extremely important to consider that observed phenotypes displayed by a snoRNA gene deletion or overexpression mutant can not only be due to the failure or a higher degree of modification but also due to the lack of the specific snoRNA itself which might function in some other biochemical context, *e.g*. as rRNA chaperone [20].

*In vitro* approaches able to find functional differences for specialized ribosomes but circumventing the intricacy of *in vivo* experiments are translation assays dedicated to test efficiency and accuracy of the synthesis of different polypeptides.

In addition it would be highly interesting to find environmental conditions that lead to changes in the ratio of unmodified and modified rRNA. In first experiments we could not detect significant differences in the extent of A100 modification in cells growing in a synthetic or a complex medium.

To enable a fast identification and quantification of eukaryotic rRNA modification heterogeneities in future, sensitive analytical tools will be necessary. Currently the most common way to detect 2′-*O*-ribose methylations is the use of reverse transcriptase, which arrests at most of the respective sites in rRNA molecules at low deoxyribonucleoside triphosphate concentrations [21]. However, in this type of analysis the band appearing on the autoradiogram represents the methylated RNA molecules, making the sensitive detection of small amounts of unmodified RNA difficult. In this regard the advantage of the DNAzyme approach [18] and the RNAse H cleavage assay [22] becomes evident, as they directly visualize the non-methylated RNA molecules. Although the DNAzyme method used here bears many advantages, it has still limited applicability with regard to different sites of analysis. This is reflected by our failure to identify a DNAzyme for nucleotide U2729 in the 25S rRNA, whose modification is also guided by snR51. After identification of *SNR18* overexpression to be able to suppress a translation termination defect, Hatin et al. [23] speculated about hypo-methylation of the target sites (A647 and C648 of the 25S rRNA) and the possibility that ribosomes carrying the respective modifications might be more efficient in translation termination. Unfortunately all six different DNAzymes we tested for cutting of rRNA isolated from a Δ*snr18* strain (CEN.MB793-6B) did not yield visible cleavage products, which made investigation of the proposed hypo-methylation hypothesis impossible up to now. Esguerra [13] who postulated in his PhD thesis the existence of differentially regulated rRNA modification patterns due to the different resistance phenotypes of snoRNA gene deletion mutants under different stress conditions, tried to detect rRNA molecules lacking the 2′-O-ribose methylations at nucleotide A796 (18S rRNA) or A1133 (25S rRNA) by the use of DNAzymes. However, although he could find the expected cleavage products with RNA samples from the respective snoRNA gene deletion mutants, they were not identified in RNA samples from wild type. Our own experiments ([18] and unpublished results) with mid to high efficiency DNAzymes showed also no significant cleavage of wild type 18S rRNA at nucleotides A974, G1126, G1271 and wild type 25S rRNA at nucleotides G1450, G2815 (numbering according to the rRNA sequence numbering used at the Saccharomyces Genome Database, www.yeastgenome.org). The absence of a visible cleavage band in the analytical gel does not prove, though, the respective nucleotide to be modified in all ribosomes of the cell culture, as the sensitivity of the DNAzyme approach is clearly limited by DNAzyme efficiency and the detection limit of ethidium bromide staining.

In the present study, we provided the highly sensitive LC-UV-MS/MS analysis as an important tool for the accurate quantification of RNA modification. Using this approach we could demonstrate the hitherto unknown heterogeneity in the 18S rRNA, showing that approximately one third of the 18S rRNA population lacks Am100. Application of this approach for other sites might help to elucidate even more heterogeneous population of ribosomes with respect to rRNA modifications.

Mutations in snoRNA genes or other RNA modification factors have been associated with Prader-Willy syndrome [24], Dyskeratosis congenita [25], Hoyeraal-Hreidarsson syndrome [26] and different forms of cancer [17], [27]–[31]. In a recent work the yeast homolog of the DKC1 gene, which is affected in many cases of Dyskeratosis congenita and Hoyeraal-Hreidarsson syndrome, was mutated, resulting in ribosomes with an overall decrease in the amount of pseudouridine residues [32]. These ribosomes were impaired in translational fidelity and IRES-dependent translational initiation in the same way as ribosomes from DKC1-deficient human cells, demonstrating ribosome-associated disease phenotypes to be the consequence of changes in rRNA modification. Our findings of heterogeneity in snoRNA-mediated RNA modifications might also have some relevance in human diseases.

## Materials and Methods

### Yeast strains and plasmids

*S. cerevisiae* strains CEN.PK2, CEN.PK2-1C and BY4741 were obtained from EUROSCARF (http://web.uni-frankfurt.de/fb15/mikro/euroscarf/). SC-F3-1 is a baker's yeast strain isolated from cider (P. Kötter, Frankfurt, unpublished). *Candida glabrata* ATCC 2001 was obtained from ATCC. For PCR-mediated deletion of the snoRNA cluster 3 which contains *SNR41*, *SNR70* and *SNR51*, the loxP-KanMX-loxP gene disruption cassette was amplified with primers Clu3-F1 (TGAGTTCCTTTTTTCTTTTTCCATTTTCTTTCTGTGTGACCAGCTGAAGCTTCGTACGC) and Clu3-R1 (CTACATAGGGTGCAAGATTAGTTAGGTTGTAGAACTAGTTGCATAGGCCACTAGTGGATCTG) and plasmid pUG6 [33] as template and used for yeast transformation [34]. After selection on YEPD plates containing 0.2 mg mL^−1^ G418, isolation of single colonies, verification of correct gene replacement by diagnostic PCR and tetrad dissection, the haploid deletion strain CEN.SO2-1A (referred to as Δ*snr51*) was received. For deletion of the *SNR18* gene the same procedure was followed with primers SNR18-F1 (GTTAACTAATAATGATTACTTTTTTTCGCTTATGTGAATGCCAGCTGAAGCTTCGTACGC) and SNR18-R1 (ATAATGATACTCTGCTCTGTGCTATCGTCAGATACTGTGAGCATAGGCCACTAGTGGATCTG). In this case the deletion cassette had to be removed before sporulation via introduction of plasmid pSH47 [33] as *SNR18* is encoded in the intron of the essential *EFB1* gene. By following this strategy, the respective haploid *snr18* deletion strain CEN.MB793-6B was viable.

For construction of plasmid pRS426-Cluster3 the snoRNA gene cluster 3 was amplified by PCR with oligonucleotides Cluster3-XhoI (ACGTCTCGAGTAAACAGTATAATACTCTAGTATGAGC) and Cluster3-EcoRI (ACGTGAATTCTGCTACTTTTCTCTTGCTGTTCTG) with chromosomal CEN.PK2-1C DNA as template, digested with *Xho*I and *Eco*RI and ligated into pRS426 [35] digested with the same enzymes.

### Cultivation, RNA isolation and polysome preparation

For isolation of RNA, yeast strains were cultivated in 5 mL medium at 30°C to an OD600 of 0.6 – 1.0. YEPD medium (1% yeast extract, 2% bactopeptone, 4% glucose) or synthetic complete medium containing 4% glucose (SCD) was used. To select for maintenance of plasmids pRS426 or pRS426-Cluster3 SCD medium lacking uracil was used. After harvesting the cells grown to exponential growth phase by centrifugation, total RNA was isolated by phenol/chloroform extraction [36] or by use of the RNeasy Mini Kit (QIAGEN).

To isolate polysomes, sucrose gradient centrifugation was performed on a 20% to 50% gradient in a buffer (20 mM HEPES pH 7.5, 10 mM KCl, 2.5 mM MgCl_2_, 1 mM EGTA and 1 mM DTT). Yeast cell lysates were prepared in the same buffer by vortexing with glass beads for 2 min at 4°C. The amount of RNA was determined at 254 nm and 20 OD254 units were loaded on the gradients. Ultracentrifugation was performed in an SW40 Ti rotor (Beckman Coulter, Inc.) for 17 h at 24,500 rpm and 4°C. Polysome fractions were pooled and precipitated with TCA, followed by RNA isolation.

### DNAzyme reactions and gel analysis

The DNAzymes used are listed in Table 1. The DNAzyme 10–23-snR51-A100 was designed to cleave 18S rRNA at nucleotide A100. DNAzymes 10–23-snR18-A649-1, 10–23-snR18-A649-2 and 10-23-snR18-A649-3 were designed for cleavage of 25S rRNA at nucleotide A649. DNAzymes 8–17-snR18-C650-1, 8–17-snR18-C650-2 and 8–17-snR18-C650-3 were designed for cleavage of 25S rRNA at nucleotide A650. The DNAzymes were purchased from Sigma-Genosys as deprotected and desalted oligonucleotides. DNAzyme-catalyzed RNA cleavage and gel analyses were performed as described previously [18].

**Table 1:** DNAzyme sequences.

DNAzyme	Sequence
10–23-snR51-A100	ATAAACGATAACTGATTTAAGGCTAGCTACAACGAGAGCCATTCGC
10–23-snR18-A649-1	GACTCCTTGGTCCGGGCTAGCTACAACGAGTTTCAAGACGGGC
10–23-snR18-A649-2	CCTTGGTCCGGGCTAGCTACAACGAGTTTCAAGACGGG
10–23-snR18-A649-3	CTTGGTCCGGGCTAGCTACAACGAGTTTCAAGACGG
8–17-snR18-C650-1	AGACTCCTTGGTCCCCGAGCCGGACGATGTTTCAAGACGGG
8–17-snR18-C650-2	CCTTGGTCCCCGAGCCGGACGATGTTTCAAGACGG
8–17-snR18-C650-3	CCTTGGTCCCCGAGCCGGACGATGTTTCAAGACG

### Mung bean nuclease protection method

The mung bean nuclease protection method was performed exactly as described previously [37]. The specific sequence of the 18S rRNA was isolated by hybridization to complementary oligo 18S-Am100 (ATCAAATAAACGATAACTGATTTAATGAGCCATTCGCAGTTTCACTGTAT). 1,000 pmoles of the synthetic deoxyoligonucleotide complementary to A76 – T125 of yeast 18S rRNA were incubated with 100 pmoles of total rRNA and 1.5 µl of DMSO in 0.3 volumes of hybridization buffer (250 mM HEPES, 500 mM KCl, pH 7.0). After hybridization mung bean nuclease and 0.02 µg/µl RNase A (Sigma-Aldrich) was added to start the digestion. Prior to the separation of the samples on a 13% polyacrylamide gel containing 7 M urea, RNA was extracted once with phenol/chloroform and subsequent ethanol precipitation. Bands were extracted using the D-Tube™ Dialyzers according to the manufacturer's protocol for electroelution (Novagen®).

### LC-UV-MS/MS quantification of 2′-*O*-methyladenosine

The rRNA fragment (final concentration 0.1 pmol/µL) was dissolved in 20 mM NH_4_OAc pH 5.3 and digested as described previously [38]. In addition, commercially available guanosine and 2′-*O*-methyladenosine (Sigma-Aldrich, Munich, Germany) were mixed in such ratios to simulate a 10%, 50% and 100% adenosine methylation turnover. An internal standard containing ^13^C labelled 2′-*O*-methyladenosine was added (10% of sample volume) to the samples for absolute quantification of adenosine ribose methylation turnover. This internal standard was received by digestion of total tRNA from *E. coli* grown in a M9 media with ^13^C-glucose as sole carbon source.

The samples were analyzed on an Agilent 1260 series equipped with a diode array detector (DAD) and Triple Quadrupole mass spectrometer Agilent 6460. A Synergy Fusion RP column (4 µm particle size, 80 Å pore size, 250 mm length, 2 mm inner diameter) from Phenomenex (Aschaffenburg, Germany) was used at 35°C. The solvents consisted of 5 mM ammonium acetate buffer adjusted to pH 5.3 using acetic acid (solvent A) and pure acetonitrile (solvent B). The elution started with 100% solvent A followed by a linear gradient to 8% solvent B at 10 min and 40% solvent B after 20 min. Initial conditions were regenerated by rinsing with 100% solvent A for 10 minutes. The flow rate was 0.35 mL/min.

The effluent from the column was first measured photometrical at 254 nm by the DAD followed by the mass spectrometer equipped with an electrospray ion source (Agilent Jet Stream). ESI parameters were as follows: gas temperature 300°C, gas flow 5 L/min, nebulizer pressure 35 psi, sheath gas temperature 350°C, sheath gas flow 12 L/min, capillary voltage 3500 V. Nitrogen was used as source and collision gas, the cell accelerator voltage was set to 2 V and the collision was induced for all analyzed with a collision energy of 15 eV. The MS was operated in positive ion mode to monitor selectively the transitions for 2′-*O*-methyladenosine (m/z: 282→136) and ^13^C-2′-*O*-methyladenosine (m/z: 293 → 141) in dynamic SRM mode with a time window of 1 minute for each analyte. For the quantification of 2′-*O*-methyladenosine, all samples were measured as described above. The data was then processed using the UV data of guanosine for quantification of injected rRNA and MS/MS data for quantification of 2′-*O*-methyladenosine.

The injected rRNA fragment was quantified using UV_254 nm_ area of the main nucleoside guanosine (9 guanosine residues per RNA fragment). Here, calibration measurements of guanosine dilutions were applied for exact quantification.

For both 2′-*O*-methyladenosine and its ^13^C-labeled derivative, the MS/MS peaks were integrated. The peaks were correlated by a previously determined response factor (RFN) (Kellner S and Helm M unpublished), which allows the calculation of the total amount of 2′-*O*-methyladenosine per sample. The raw data and calculation is shown in the supporting data S1.

## Supporting Information

Data S1 Calculation of 2′-O-methyladenosine (Am) content in injected rRNA fragment including raw data.
(DOC)
