# Supplementary material for: Partial Methylation at Am100 in 18S rRNA of Baker's Yeast Reveals Ribosome Heterogeneity on the Level of Eukaryotic rRNA Modification
Source: PLoS One. 2014 Feb 28;9(2):e89640. doi: 10.1371/journal.pone.0089640 (PMC3938493; doi:10.1371/journal.pone.0089640)

**Supporting data S1**

**Calculation of 2´-*O*-methyladenosine (Am) content in injected rRNA fragment**

Raw data:

|  | Precursor Ion | Product Ion | Retention time | Peak area |
| --- | --- | --- | --- | --- |
| Am | 282 | 136 | 15.984 | 358369 |
| Am ^13^C | 293 | 141 | 15.979 | 8940 |

For calculation of the amount of injected Am, the following equation is used


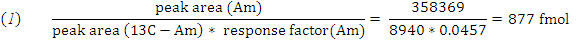


The amount of injected Am is correlated to the amount of injected RNA. This amount is received by analysis of the guanosine peak in the 254 nm chromatogram. The peak area for the wt sample for this measurement is 155.4. This signal contains the signal from the rRNA guanosine and the ^13^C guanosine signal from the internal standard (ISTD). The signal intensity for the added internal standard is 129.82. Therefore, the share of guanosine signal arising from the rRNA fragment is


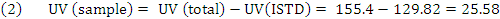


Now, the amount of injected guanosine in pmol can be calculated by using a guanosine calibration factor (2.21).


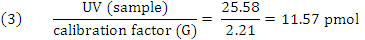


To assess the amount of RNA, the number of guanosines of the analyzed RNA fragment must be known. Here, 9 guanosine nucleosides are contained in the fragment sequence.


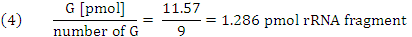


Finally, the amount of Am can be correlated with the amount of rRNA fragment:


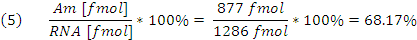

Supplement: Data S1 — Calculation of 2′-O-methyladenosine (Am) content in injected rRNA fragment including raw data. (DOC) [file pone.0089640.s001.doc]
